# Monoclonal Antibodies Directed Against IL-5 in the Treatment of Pediatric Asthma

**DOI:** 10.3390/cells15141246

**Published:** 2026-07-10

**Authors:** Valentina Fainardi, Roberta Carbone, Enrico Vito Buono, Marialaura Menzella, Carlo Caffarelli

**Affiliations:** Pediatric Clinic, Department of Medicine and Surgery, University of Parma, 43126 Parma, Italy; valentina.fainardi@unipr.it (V.F.); roberta.carbone2@unipr.it (R.C.); enricovito.buono@unipr.it (E.V.B.); marialaura.menzella@unipr.it (M.M.)

**Keywords:** pediatric severe asthma, severe treatment-resistant asthma, eosinophils, interleukin-5, mepolizumab, benralizumab, biologics

## Abstract

Severe treatment-resistant asthma (STRA) in children is often sustained by type 2 inflammation and eosinophil-dependent airway disease that persists despite optimized inhaled therapy and the mitigation of modifiable factors. This review summarizes the clinical and translational evidence on monoclonal antibodies targeting the interleukin-5 (IL-5) axis (anti-IL-5 and anti-IL-5Rα) available in pediatric severe asthma. PubMed/MEDLINE was searched up to January 2026 for English-language studies in patients aged 0–18 years addressing mepolizumab and benralizumab, including randomized trials, high-quality observational studies, meta-analyses, and international guidance. Mepolizumab has the most robust pediatric data, showing consistent reductions in exacerbations and blood eosinophils, and improvements in symptom control and quality of life, with safety broadly comparable to adults. The pediatric evidence for benralizumab is more limited but shows rapid eosinophil depletion, improved outcomes in selected children, and acceptable safety; further trials are ongoing. Overall, IL-5–directed biologics represent a key add-on option for carefully selected children with severe eosinophilic asthma, while pediatric-specific predictors of response, comparative effectiveness, and standardized long-term monitoring and stopping criteria remain priorities.

## 1. Introduction

Asthma is a chronic disease characterized by airway inflammation, bronchial hyperresponsiveness, and reversible air flow obstruction resulting in variable respiratory symptoms [1]. Worldwide, about 10% of children are affected by this condition. Most of them have atopic asthma with aero-allergen sensitization and eosinophilic airway inflammation. Low doses of inhaled corticosteroids (ICSs) are usually successful in achieving good symptom control and minimizing acute attacks in the majority. However, it is estimated that 2–5% of children have problematic severe asthma with poor control of symptoms despite maximum asthma therapy and a higher risk of exacerbations and asthma-related deaths [2,3]. After the appropriate work-up, children with problematic severe asthma can be classified into difficult asthma and severe treatment-resistant asthma (STRA). Children with difficult asthma include most children with problematic severe asthma who have a steroid-sensitive disease which improves with the adherence to daily inhaled corticosteroids and after having removed modifiable factors like suboptimal adherence, poor inhalation technique, adverse environmental exposures, and psychosocial factors [4]. On the other hand, children with persistent symptoms despite having addressed these modifiable factors are instead described as having STRA. As described by the European Respiratory Society (ERS) and the American Thoracic Society (ATS), STRA is defined as asthma that remains uncontrolled despite treatment with high-dose ICSs plus a second controller (and/or systemic corticosteroids) and after optimization of modifiable factors like adherence, inhaler technique, environmental exposures, and comorbidities [5].

Uncontrolled severe asthma affects daily activities including a limitation in physical exercise, causes night awakenings and missing days of school, and can result in poor quality of life and frequent healthcare use [6].

Furthermore, children with severe asthma are at risk of airway remodeling and a decline in lung function, with progressive effects that can track into adulthood, increasing the risk of chronic obstructive pulmonary disease (COPD) [7].

When asthma remains uncontrolled despite attempts to address modifiable factors and maximal therapy, adding biologic therapies is the preferred option [8,9]. The advent of monoclonal antibodies (mAb) directed towards different targets of asthma inflammatory pathways represented a breakthrough advance in the treatment of severe asthma. Omalizumab is directed against IgE, mepolizumab, benralizumab, reslizumab and depemokimab against interleukin (IL)-5 or IL-5 receptor subunit alpha (IL-5Rα), dupilumab against IL-4Rα, and tezepelumab against thymic stromal lymphopoietin (TSLP) (Table 1) [10]. The choice of biologic depends on the asthma phenotype/endotype described by specific biomarkers such as the blood eosinophil count, fractional exhaled nitric oxide (FeNO), and total/allergen-specific IgE [11].

This review summarizes the evidence on biologic therapies for severe pediatric asthma directed against IL-5. A structured literature search was conducted in PubMed and Scopus to identify studies evaluating the use of anti-IL-5 biologics in children and adolescents with severe asthma. The searches were performed using the terms “severe asthma” combined with “mepolizumab”, “benralizumab”, or “depemokimab”. Searches were restricted to English-language articles involving pediatric populations (0–18 years) in PubMed and to English-language articles indexed as ‘child’ or ‘adolescent’ in Scopus. Duplicates were removed before screening. Because this article was designed as a narrative review, the structured database search was complemented by manual searches of the reference lists of eligible articles and landmark publications. Additional studies considered clinically or scientifically relevant by the authors were included to provide a comprehensive overview of the current evidence, particularly regarding pivotal clinical trials, pediatric extension studies, long-term follow-up data, and recent regulatory developments. The search strategy and the study selection process is available in the Supplementary Materials.

## 2. Asthma Pathogenesis

Asthma is a heterogeneous disease that encompasses various endotypes and phenotypes.

In children, the disease has been classified in two main endotypes according to airway inflammation: type 2 (T2)—high (eosinophilic inflammation) asthma or T2—low (neutrophilic/paucigranulocitic inflammation) [12,13,14].

The majority of pediatric asthma shows a T2—high endotype with raised levels of blood (eosinophil count ≥ 150 cells/µL) and airway eosinophils (>1–3% in the induced sputum) [15]. High blood eosinophils correlate with exacerbation risk, especially if combined with elevated FeNO (≥20 ppb in children) [16].

The onset of this inflammatory pathway begins when allergens, microbes, smoke, or other pollutants attack the epithelial barrier resulting in epithelial damage. Allergens and microbes are presented to naïve CD4 + T cells by antigen-presenting cells such as dendritic cells or macrophages. Naïve CD4 + T cells then switch to T helper (Th)-2 lymphocytes with the activation of the Th2 pathway. In response to the aforementioned triggers, including pollutants as well, the damaged epithelium releases epithelial-derived alarmins such as thymic stromal lymphopoietin (TSLP), IL-25, and IL-33, known as innate cytokines. These cytokines activate the innate lymphoid cells type 2 (ILC-2) and adaptive immunity (Th2), leading to Th2 polarization and eosinophilic inflammation. In addition, IL-33 can influence local tissue eosinophil progenitor cell development, via the release of IL-5 from eosinophil progenitors, and is a potent activator of mature eosinophils [17].

Both ILC-2 and Th2 cells produce IL-4, IL-5, and IL-13 and both drive eosinophil activation and recruitment through an IL-5-mediated pathway. IL-5 acts by binding the alpha-chain of the IL-5 receptor expressed on mature eosinophils and basophilic cells. The intermediate complex formed after the binding of IL-5 with IL-5Rα recruits the receptor (CD131), forms a ternary complex, and activates the JAK/STAT signaling cascade, eventually promoting eosinophil generation and differentiation from CD34+ progenitors [18,19,20,21]. Eosinophil recruitment is then sustained by CCL26 and IL-5 that induce the chemotaxis of eosinophils from the blood to the tissues. IL-5 also stimulates eosinophil activation and degranulation with the release of inflammatory cytokines that cause the upregulation of both innate and adaptive immune responses. Eosinophilic inflammation is generally mediated by the releasing of eosinophil cationic protein, prostaglandins, leukotrienes, major basic protein (MBP), eosinophil-derived neurotoxin, and eosinophil peroxidase. The activation of eosinophils by IL-5 is associated with the reduced expression of CD62L at the cell surface (CD62LLow eosinophils). These cells are found in T2—high inflammatory tissue such as nasal polyp tissue and in patients with asthma [22,23,24]. Taken together, these mediators contribute to asthma exacerbation, promoting airway hyperresponsiveness and smooth muscle contraction, mucous secretion, and vasodilation. Persistent eosinophilic inflammation ultimately drives airway epithelial remodeling, a process further amplified by IL-5 together with transforming growth factor (TGF)-beta [25].

Furthermore, about 80% of asthmatic children has a T2 allergic endotype with high levels of specific IgE produced by B cells stimulated by IL-4 and IL-13 [11,13]. Specific IgEs trigger basophil and mast cell degranulation with histamine, prostaglandin, and leukotriene release, which further contribute to asthma with bronchoconstriction, mucus production, and edema [1].

Non-T2 asthma is more common in adults than in children and it has been linked with Th17/Th1 activation and with neutrophilic or paucigranulocytic airway inflammation with the release of proinflammatory cytokines such as IL-17, IL-6, and tumor necrosis factor-α (TNF-α). This type of asthma usually responds poorly to steroids [12,13].

However, the two definitions of T2 and non-T2 asthma can be misleading since the two endotypes can manifest with similar clinical symptoms and therefore phenotypes. Furthermore, these features can vary over time and overlap, as demonstrated by long-term longitudinal follow-ups [26].

## 3. STRA Phenotype and IL-5

Children with STRA have persistent symptoms despite maximal treatment and despite having addressed modifiable factors like suboptimal adherence, poor inhalation technique, persistent adverse environmental exposures, and psychosocial factors. The patients are mainly males with atopy and multiple sensitizations—they show eosinophilic airway inflammation and airway obstruction with remodeling [27,28].

In STRA patients, type 2 inflammation is driven both by Th2 and by ILC2 [29]. Th2 and ILC2 can secrete IL-5, a key mediator of eosinophil proliferation, activation, and survival, and is, therefore, central to eosinophilic airway inflammation [30].

In severe asthma, the airway accumulation of eosinophils is crucial in the pathogenesis of T2 inflammation since eosinophils mediate airway obstruction and bronchial hyperresponsiveness. The local release of chemokines, leukotrienes, growth factors, and metalloproteinases by activated eosinophils contribute to persisting inflammation and airway remodeling [20]. The link between elevated blood and sputum eosinophil counts and an increased risk of asthma exacerbations in children has been documented [31,32,33].

In severe asthma, treatment with biologics targeting eosinophils has been associated with reduced asthma exacerbations triggered by viral infections since the reduction in eosinophils may improve epithelial barrier function, decrease airway injury, and potentially increase the immune response against respiratory virus infections [25,34].

At present, four anti-IL-5 monoclonal antibodies have been produced to block the IL-5 pathway, thereby reducing eosinophil activation, survival, and trafficking. Thus far, regulatory agencies have approved, in children, mepolizumab and, in adolesecents (>12 years), benralizumab and depemokimab; reslizumab is currently restricted to adults. For this reason, this review will summarize the evidence in children only for mepolizumab, benralizumab, and depemokimab.

Mepolizumab is a monoclonal IgG targeting IL-5 and inhibiting its interaction with the IL-5Rα subunit of the specific receptor. It has been approved for severe asthma in children aged ≥6 years, eosinophilic nasal polyps, and eosinophilic granulomatosis with polyangiitis (EGPA) in children aged ≥6 years [35].

**Figure 1:**
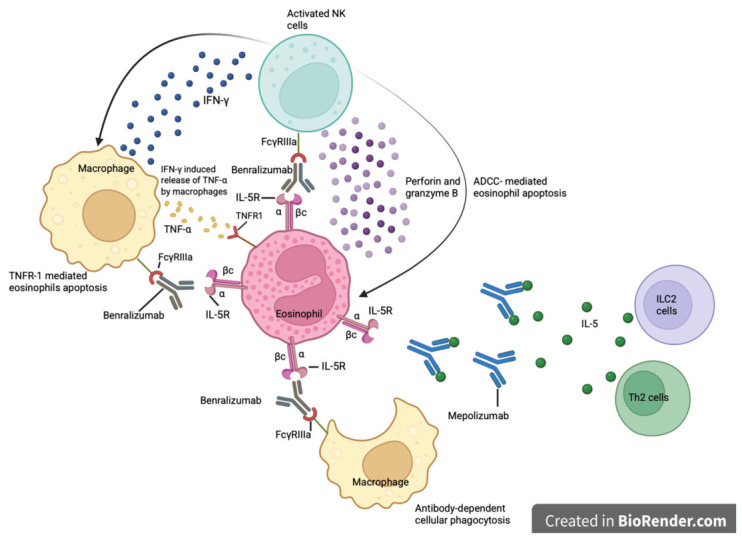
Mechanism of action of mepolizumab and benrelizumab. Figure generated with Biorender (https://www.biorender.com/). NK, natural killer; IFNγ, interferon gamma; TNFR-1, tumor necrosis factor receptor; FcγRIII, Fc gamma receptor III; ILC, innate lymphoid cells; ADCC, antibody-dependent cellular cytotoxicity; Th, T helper.

Benralizumab is a monoclonal IgG that binds the IL-5Rα receptor on the surface of eosinophils and basophils, inducing apoptosis through antibody-dependent cellular cytotoxicity. It produces rapid and near-complete eosinophil depletion. The binding to IL-5Rα blocks all pathways of eosinophil activation. It has been approved for severe asthma in children ≥6 years. Figure 1 depicts the mechanism of action of mepolizumab and benralizumab on the IL-5 pathway [19,36].

Resliizumab is a monoclonal IgG4 that targets IL-5 and blocks the IL-5Rα receptor. It has been approved for severe asthma in adults [37].

Depemokimab binds IL-5. It is approved for severe asthma in adolescents (≥12 years) and adults. It is administered subcutaneously every 6 months. Its evidence in asthma and in nasal poliposis are limited to four replicate Phase III clinical studies (SWIFT-1 and SWIFT-2 for asthma and ANCHOR-1 and ANCHOR-2 for nasal poliposis) [38,39].

## 4. Evidence of Mepolizumab and Benralizumab in Pediatric Asthma

### 4.1. Mepolizumab

Mepolizumab is a large-molecular-weight monoclonal antibody (149.2 kiloDaltons) that selectively blocks IL-5; it does not interfere with the other cytokine networks involved in eosinophil activation.

The effect of mepolizumab is the interruption of eosinophil activity affecting their proliferation, differentiation, mobilization, activation, recruitment, and survival as demonstrated by the reduction in these cells in sputum and blood [40].

The blood eosinophil count and asthma exacerbations in the previous year are the variables most associated with the mepolizumab response [41].

According to GINA, mepolizumab is approved as a Step 5 add-on for severe eosinophilic asthma in children ≥ 6 years with blood eosinophils ≥ 150 cells/µL and recurrent exacerbations despite high-dose ICSs plus at least one additional controller [8]. There are six trials conducted in children that supported this clinical indication (Table 2).

In children aged 6 years and above, mepolizumab has been also approved for eosinophilic granulomatosis with polyangiitis (EGPA). In adults, mepolizumab has been also approved for severe chronic rhinosinusitis with nasal polyps and for hypereosinophilic syndrome (HES).

#### 4.1.1. Clinical Effects in Severe Asthma

In children with severe eosinophilic asthma who experienced at least two exacerbations in the previous year, treatment with mepolizumab is associated with a reduction in exacerbations [41,42,43,44,45], symptoms [42,43,44,46], and eosinophils [41,42,44,45], and an increase in quality of life [42,43,44,46] and lung function [42].

The MENSA (Mepolizumab as Adjunctive Therapy in Patients with Severe Asthma) study was a 32-week trial that included 576 patients (25 adolescents aged 12–18 yrs). The subcutaneous administration of mepolizumab decreased blood eosinophil counts by 86%, increased FEV_1_ by about 100 mL, and reduced exacerbations by 53%. An overall improvement in symptoms and quality of life was observed [42].

The SIRIUS (Steroid Reduction with Mepolizumab Study) included 2 adolescents among 135 subjects on continuous treatment with oral prednisone for at least 6 months. Every 4 weeks, the steroid dose was reduced by 1.25–10 mg for a period of 16 weeks; at the follow-up visit at week 32, those who received subcutaneous mepolizumab had significantly greater reductions in the maintenance oral glucocorticoid dose (up to 50%), fewer exacerbations (32%), better quality of life, and better asthma control [43].

In a further multicenter, double-blind placebo-controlled trial study, the DREAM (Dose Ranging Efficacy And safety with IV Mepolizumab) study, only one adolescent was included, limiting the interpretability and the application of the results to pediatric age. The trial had a length of 52 weeks and showed a reduction in blood eosinophils and a significant reduction in asthma exacerbation, especially in the arm treated with the highest dosage of 750 mg iv (range 39–52%). The most common adverse effects were nasopharyngitis, headache, and local reactions in the site of injection [41].

The MUSCA study explored, in a 24-week trial including nine adolescents, the efficacy of subcutaneous 100 mg mepolizumab on health-related quality of life and demonstrated improved symptoms and quality of life [46].

In a post hoc meta-analysis of adolescents included in the MENSA and MUSCA trials, there was a 40% reduction in the annual rate of exacerbations, a similar percentage observed in adults.

Taken together, these four studies included a total of 34 adolescents. Despite this low number that may prevent the form generalization of the results, a Phase III study showed the comparable efficacy and safety of mepolizumab between adolescents with severe eosinophilic asthma and the overall population [47].

More recently, data from Phase III trials in adults (n = 1841) and adolescents aged 12–17 years (n = 37) with severe asthma, from the open-label trial in children aged 6–11 years (n = 36), and from the trial in children aged 2–11 years (n = 32) and adolescents aged 12–17 years (n = 27) with eosinophilic esophagitis were extrapolated to further support the pediatric indication of mepolizumab in severe asthma. The study concluded that the pharmakokinetic and pharmacodynamic effects, and efficacy found in adults could reliably predict pediatric outcomes [48].

An important study performed in children was the 12-week trial by Gupta et al. on the use of subcutaneous mepolizumab with both a dosage of 40 and 100 mg administered every 4 weeks in 36 children aged 6 to 11 years with severe eosinophilic asthma. Blood eosinophils were reduced by more than 80% and the symptoms improved. No difference was found in FEV_1_ [33]. In the 52 weeks of follow-up, patients had a reduction in exacerbations of 69% and more than 50% of children reported an improvement in quality of life [49].

More recently, the Mechanisms Underlying Asthma Exacerbations Prevented and Persistent With Immune-Based Therapy: A Systems Approach Phase 2 (MUPPITS-2) trial, a randomized double-blind placebo-controlled trial including 290 children from minority ethnic groups in socially deprived areas in the USA aged 6–17 years, reported a significant reduction in asthma attacks over 12 months, much more evident in adults (50%) than in children (27%) [45]. There might be several reasons to justify this difference in the exacerbation rate between adults and children. Some children still experienced exacerbations maybe because exacerbations were related to pollution or second-hand smoke rather than related to the virus. Furthermore, the included subjects may not have been the ideal targets for mepolizumab due to the low number of eosinophils required to be admitted in the trial. The threshold of 150 blood eosinophils can be very low to select those subjects with true eosinophilic inflammation, since, in children, these cells can be much higher than in adults and the level can also vary within a short period of time [52,53]. In addition, as demonstrated, younger children can have lower levels of IL-5 in bronchoalveolar lavage, and, therefore, mepolizumab may not be as efficacious in young children as in adults [54]. In the MUPPITS trial, a significant reduction in blood and nasal eosinophils was reported while no difference was noted in FEV_1_ [45].

The mechanisms involved in asthma exacerbations occurring in these patients despite the treatment with mepolizumab were further analyzed with different results.

The Sputum CytOf sUbsTudy (SCOUT) was a sub-study of MUPPITS-2 that identified, in the induced sputum of the participants, three eosinophil populations with a different CD62L expression. Children on mepolizumab experiencing exacerbations showed higher proportions of eosinophils expressing high levels of CD62L (CD62L intermediate/high), which showed upregulated activation markers (CD69 and CCR3), increased eosinophil peroxidase, and an increased secretion of IL-5, IL-13, and TNF-α. The persistence of these activated eosinophils suggests a resistance to mepolizumab and may explain the residual susceptibility to asthma attacks of some patients [55].

Eosinophils expressing low levels of CD62L (CD62L low) correlate with the severity of asthma and are usually reduced by mepolizumab [56].

A further secondary analysis of the MUPPITS-2 trial was conducted in 108 participants with exacerbations. The transcriptomic analysis of their nasal lavages obtained during 176 acute respiratory events showed that exacerbations were mainly caused by non-Th2 inflammation driven by epithelial activation and the secretion of IL-33, the hypersecretion of mucus, remodeling, and the activation of macrophages. These results may reveal that, in some subjects, there are other pathways of inflammation beyond IL-5 that can contribute to residual exacerbations in children treated with mepolizumab [50]. Although conducted in adults, this hypothesis was also suggested by the MEX study. This was a prospective observational study including 145 patients with severe eosinophilic asthma treated with mepolizumab where half of the exacerbations occurring during treatment with mepolizumab were neutrophilic. The eosinophilic exacerbation was characterized by a high sputum eosinophil count, higher blood eosinophil counts, and lower FEV_1_ compared to the neutrophilic phenotype. The correlation between blood eosinophils and lung function was previously suggested by Hancox et al. who assessed lung function at 21, 26, 32, and 38 years in 971 adults with and without asthma. The authors found that higher blood eosinophils were associated with a lower FEV_1_ and enhanced decline in lung function, independently of asthma and smoking [57].

Data on the use of biologics in severe asthma in pediatric age have been recently collected in a European study. Of the 250 children enrolled in the Severe Paediatric Asthma Collaborative in Europe (median age 13.2 years), 21.6% (n = 54) were on mepolizumab. Most had a good quality of life, 70% had good asthma control, but 83% experienced at least one exacerbations in the last year [51].

Retrospective data on a real-world study conducted in the USA in 580 patients aged 6–17 years with severe asthma (47% were aged 6–11 years) and treated with mepolizumab showed a 24% reduction in OCS, a reduction of 34% in asthma exacerbations [58].

In a retrospective analysis of 16 patients (aged 7–17 years) comparing healthcare resource utilization in the 12 months before and after starting mepolizumab, hospital admissions decreased by 67% [59], suggesting an overall improvement in asthma exacerbations.

The cessation of mepolizumab is associated with an increase in blood and sputum eosinophil count, followed by a rise in symptoms and exacerbations at 3–6 months [60,61].

There is an ongoing trial on the efficacy of mepolizumab compared with omalizumab in young people with true STRA and refractory difficult asthma. One goal is the assessment of the optimal biomarkers that might predict a clinical response for each biologic and the evaluation of activated eosinophils rather than the number of blood eosinophils to determine the likely response to therapy [62].

The clinical effects of mepolizumab are described in Table 3.

#### 4.1.2. Pharmacokinetics, Pharmacodynamics, and Metabolism of Mepolizumab

The routes of administration (i.e., intravenous, subcutaneous, and intramuscular) and a range of dosages (from a minimum of 0.05 to a maximum of 10 mg/kg) were reviewed [63].

Overall, mepolizumab pharmacokinetics is linear, dose-dependent, and time-independent.

Following both IV and SC administration, mepolizumab was quantifiable for up to 16 weeks.

Mepolizumab shows similar pharmacodynamics after intravenous, subcutaneous, or intramuscular administration. The reduction in the blood eosinophil count correlated with the dose administered.

In all studies, the pharmacokinetics and pharmacodynamics were not affected by age and sex [63].

More recently, population pharmacokinetic analyses from the Phase III mepolizumab clinical development program demonstrated no clinically meaningful effect of age on mepolizumab pharmacokinetics, with adolescents exhibiting a drug clearance comparable to that observed in adults. In contrast, a dedicated pediatric study in children aged 6–11 years showed a lower apparent clearance and, consequently, higher systemic exposure compared with adults [44,47].

#### 4.1.3. Safety

The follow-up of MENSA and SIRIUS studies resulted in a further study called the COSMOS study on the long-term efficacy and safety of mepolizumab in the 26 pediatric patients. Adolescent patients had been treated with mepolizumab (100 mg subcutaneous) for a median of 12.1 months (range 2–20 months). The adverse events reported for adolescents was similar to the rest of population and were nasopharyngitis, sinusitis, and asthma [64].

In the pediatric study by Gupta et al., 27% of the 30 children included had headache, abdominal pain, and pyrexia as the most common side effects [49].

A multicenter, Phase IIIb safety, open-label extension study of multiple studies published between 2015 and 2022 demonstrated that, in 24 patients aged 6–17 years, mepolizumab is overall safe in the treatment of eosinophilic asthma in addition to the standard of care. Serious adverse events were uncommon, mainly mild infections, and none were attributed to treatment. No anaphylaxis was reported [65].

### 4.2. Benralizumab

Benralizumab binds to IL-5Rα via its fab fragment. The Fc region of benralizumab, the CH2 domain, via FcγRIIIa receptors (also known as CD16), interacts with natural killer (NK) cells leading to the release of the apoptotic proteins like granzymes and perforin, amplifying Antibody-Dependent Cellular Cytotoxicity (ADCC) activity up to 100 times. Activated NK cells release interferon (IFN)-γ, which stimulates TNF-α secretion from macrophages. TNF-α then binds to the TNF receptor 1 expressed on eosinophils, inducing apoptosis and, consequently, enhancing Antibody-Dependent Cellular Phagocytosis (ADCP). Macrophages are also activated for phagocytosis via FcγRIIIa receptors. The final result is the complete depletion of eosinophils [66]. Benralizumab has been approved for uncontrolled severe asthma in adults and children ≥6 years and is also indicated as an add-on treatment for adult patients with relapsing or refractory eosinophilic granulomatosis with polyangiitis. The inclusion criteria depend on countries. For example, in Italy, benralizumab is considered when the patient has blood eosinophil count ≥300 cells/µL with ≥2 exacerbations requiring OCSs in the prior 12 months OR ongoing maintenance OCS ≥5 mg prednisolone/day within the past 12 months [37,67]. In the UK, the biologic can be prescribed when the patient has blood eosinophil count ≥300 cells/µL with ≥4 exacerbations requiring OCSs in the prior 12 months OR ongoing maintenance OCS ≥5 mg prednisolone/day within the past 6 months OR ≥400 cells/µL with ≥3 exacerbations requiring OCSs in the prior 12 months [68].

The recommended dose of benralizumab is 30 mg by subcutaneous injection every 4 weeks. For children, the dose is weight-based (30 mg for children ≥35 kg and 10 mg for children <35 kg). The treatment is administered every four weeks for the first three doses and then continued with maintenance doses every eight weeks [37].

Last summer 2025, an investigation plan has been submitted to EMA to assess the efficacy in children with hypereosinophilic syndrome (HES) [69].

#### 4.2.1. Clinical Effects in Severe Asthma

Data on the efficacy of benralizumab in children are limited. Trials including children are described in Table 4.

The Food and Drug Administration (FDA) authorization of benralizumab for children 6–11 years was supported by the TATE study, a 48-week, open-label trial assessing the pharmacokinetics, pharmacodynamics, and safety in 28 pediatric patients with severe eosinophilic asthma (blood eosinophil ≥ 150 cells/µL) and a history of at least two exacerbations requiring systemic corticosteroids and/or hospitalization within the previous 12 months despite treatment with inhaled corticosteroids. Participants < 35 kg were administered benralizumab 10 mg subcutaneously, while those ≥35 kg received benralizumab 30 mg subcutaneously at baseline and at weeks 4, 8, 16, 24, 32, and 40. The study reported an approximate 50% reduction in exacerbations and improved symptom control with both dosages and an increase in quality of life and FEV_1_ [70]. Safety was favorable: the most common adverse events were mild upper-respiratory infections and injection-site reactions. Anti-drug antibodies occurred in 14.3% of participants without an apparent impact on efficacy or safety [70].

Two Phase 3 trials (SIROCCO and CALIMA) included 108 adolescents between 12 and 17 years (SIROCCO 53 patients and CALIMA 55 patients) [71,72].

Of these patients, 46 patients received the placebo, 40 received benralizumab for 3 doses every 4 weeks and after every 8 weeks, and 22 received benralizumab every 4 weeks. In SIROCCO trial, the asthma exacerbation rate was reduced by 45% with benralizumab administered for 4 weeks and by 51% with 8-weekly doses when compared with the placebo [71].

In the CALIMA trial, the exacerbation rate reduction was 36% in 4-weekly regimen and 28% in 8-weekly regimen compared with the placebo [72]. Improved results, such as fewer exacerbations, higher FEV_1_, better asthma control, and an enhanced quality of life, were observed in individuals with elevated Th2 inflammatory markers [75].

Additional pediatric trials are ongoing, enrolling children aged 6–18 years.

The first is BRISOTE, an ongoing Phase 4 study which will assess the efficacy and safety of adding a fixed 30 mg dose of benralizumab, given subcutaneously every 4 weeks for the first three doses and then every 8 weeks, in children aged ≥12 years with a history of eosinophilic asthma who remained uncontrolled on medium-dose ICS–LABA therapy, with or without additional controller medications (excluding oral corticosteroids). The trial is expected to terminate in November 2027 [76].

The second study ongoing is DOMINICA, a Phase 3 study which aims to evaluate the effectiveness and safety of benralizumab in pediatric patients aged 6–18 with severe eosinophilic asthma, and to generate additional evidence to help guide treatment in children. The primary endpoint of the DOMINICA study is time to first protocol-defined asthma exacerbation, defined as a worsening of asthma that requires a medical intervention. Findings from the DOMINICA study may support a new treatment option for children with severe eosinophilic asthma and will add to the evidence from the CALIMA, SIROCCO, and TATE studies [77].

#### 4.2.2. Pharmacokinetics, Pharmacodynamics, and Metabolism of Benralizumab

After subcutaneous administration, benralizumab is absorbed slowly and follows linear, dose-proportional pharmacokinetics that are well-described by a two-compartment model with first-order absorption and elimination. Benralizumab shows pharmacokinetic characteristics typical of IgG monoclonal antibodies, with linear systemic clearance and a terminal half-life of approximately 2–3 weeks; there is no evidence of metabolism via hepatic enzymatic pathways [78].

#### 4.2.3. Safety

In the BORA trial, 72 adolescent patients between 12 and 17 years old who had completed the SCIROCCO and CALIMA trials continued receiving subcutaneous benralizumab every 4 weeks or every 8 weeks, for a total of 108 weeks. The patients who had received the placebo in the two previous trials were randomized to receive benralizumab every 4 or 8 weeks in this trial. The main objective was to assess the safety and tolerability of the two benralizumab dosing regimens. Across all groups, the most frequently reported adverse events were viral upper respiratory tract infections and asthma exacerbations. The most common serious adverse events included asthma worsening, pneumonia, and bacterial pneumonia. The proportions of patients experiencing any treatment-emergent adverse event, any serious adverse event, or any adverse event leading to discontinuation during BORA were comparable between those initially treated with benralizumab and those given placebo, as well as between the different benralizumab dosing regimens [73].

The ALIZE study showed that benralizumab 30 mg administered every 4 weeks did not reduce the antibody response to influenza vaccination in adolescents and young adult patients with moderate-to-severe asthma [74].

### 4.3. Depemokimab

Depemokimab is a next-generation, ultra-long-acting anti-IL-5 monoclonal antibody developed for the treatment of severe eosinophilic asthma. Through high-affinity binding to IL-5, it inhibits eosinophil proliferation and survival, resulting in the sustained suppression of eosinophilic inflammation. Structural modifications in its Fc region prolong systemic exposure and enable a convenient dosing regimen of one subcutaneous injection every six months, potentially improving treatment adherence compared with currently available anti-IL-5 therapies.

It is approved for severe asthma in adolescents (≥12 years) and adults.

#### 4.3.1. Clinical Effects in Severe Asthma

The evidence in asthma is limited to two Phase III clinical studies (SWIFT-1 and SWIFT-2) [38,39], which enrolled 762 patients aged ≥12 years with severe eosinophilic asthma inadequately controlled, despite high-dose inhaled corticosteroids and additional controller therapy. Across the two studies, depemokimab significantly reduced the annualized rate of clinically significant exacerbations by 58% and 48%, respectively, compared with placebo. Moreover, fewer patients experienced exacerbations requiring hospitalization or emergency department visits. Treatment induced a rapid and sustained reduction in blood eosinophil counts, with decreases of approximately 79% relative to placebo maintained through week 52. Improvements in health-related quality of life, asthma control, and lung function were not significant compared to placebo. Although the number of the included adolescents was limited (n = 30), a pooled analysis showed a 43% reduction in clinically significant exacerbations compared with placebo, suggesting that the clinical benefit observed in adults is also present in this age group [38,39]. No data are currently available for children younger than 12 years.

#### 4.3.2. Pharmacokinetics, Pharmacodynamics, and Metabolism of Depemokimab

Depemokimab exhibits approximately dose-proportional pharmacokinetics following subcutaneous administration and was specifically engineered to achieve prolonged systemic exposure. The molecule incorporates YTE amino acid substitutions within the Fc region, enhancing binding to the neonatal Fc receptor (FcRn) and extending its terminal half-life to approximately 39–53 days, considerably longer than conventional IgG1 antibodies. Following a 100 mg subcutaneous dose, peak plasma concentrations are reached after a median of 8–14 days, and negligible accumulation is observed with repeated administration every six months. Depemokimab is catabolized through ubiquitous proteolytic pathways into small peptides and amino acids and is not eliminated through renal excretion or hepatic metabolism. Pharmacodynamically, depemokimab binds human interleukin-5 (IL-5) with picomolar affinity, preventing its interaction with the IL-5 receptor α on eosinophils and thereby inhibiting eosinophil maturation, activation, and survival. This results in the rapid and sustained suppression of blood eosinophils, with reductions of approximately 54% within 24 h after administration and up to 79–85% during long-term treatment. Population pharmacokinetic analyses demonstrated no clinically relevant effects of age, sex, ethnicity, renal function, or hepatic function on drug exposure. In adolescents aged 12–17 years, the pharmacokinetic parameters were comparable to those observed in adults, supporting the use of the same dosing regimen across these age groups.

Pharmacokinetic analyses further demonstrated comparable drug exposure between adolescents and adults, supporting the use of the same 100 mg dose administered every six months in patients aged 12 years and older [39,79].

#### 4.3.3. Safety

Depemokimab has shown a favorable safety profile in patients with severe eosinophilic asthma. In the Phase III SWIFT trials, adverse events were generally mild to moderate and comparable to placebo. The most common treatment-related events were injection-site reactions (1–2%) and mild systemic reactions such as headache, fatigue, and rash. Anti-drug antibodies were detected in approximately 8–9% of patients, while neutralizing antibodies occurred in less than 1% and had no apparent impact on efficacy or safety. Importantly, no new safety signals were identified in adolescents, whose safety profile was similar to that observed in adults [38,39].

## 5. Conclusions

Biologics targeting the IL-5 axis represent a major advance for patients with severe eosinophilic asthma, offering clinically meaningful reductions in exacerbations and systemic corticosteroid exposure and improving symptom control and quality of life when added to optimized standard therapy.

Before initiating a biologic, careful pheno- and endotyping is essential, including the confirmation of the diagnosis, the assessment of comorbidities and modifiable factors (adherence, inhaler technique, and exposures), and the use of available biomarkers (blood eosinophils, FeNO, and IgE/allergen sensitization where relevant), alone or in combination, to align treatment choice with the underlying endotype.

In STRA, the basis of treatments like adherence to treatment, exposure to allergens, or inhalation technique must be optimized. If children remain uncontrolled despite comprehensive multidisciplinary management, biologics can be considered. When used appropriately, biologics can substantially reduce the attack frequency and severity in selected children, including those with STRA who are at high risk of life-threatening exacerbations.

Despite the low numbers of children and adolescents included in the trials, mepolizumab has the strongest pediatric evidence among IL-5-directed therapies and it is considered effective for children with severe eosinophilic asthma. However, some studies report that a subset of patients suffer from residual exacerbations despite the treatment with mepolizumab suggesting that some might have other inflammatory pathways and therefore need further investigations.

Although mepolizumab demonstrated comparable pharmacokinetic and pharmacodynamic profiles across age groups, emerging evidence suggests that their clinical effects may not be entirely age-independent. Population pharmacokinetic analyses showed similar clearance in adolescents and adults, while children aged 6–11 years exhibited a lower apparent clearance and higher systemic exposure without relevant safety concerns. Nevertheless, the clinical response observed in pediatric studies appears more heterogeneous than that reported in adults. In the MUPPITS-2 trial, for example, mepolizumab had a smaller effect on asthma exacerbations in children and adolescents compared to the effect reported in adults. Explanations can be a different underlying asthma endotype with a greater contribution of non-type 2 inflammatory pathways, alternative inflammatory pathways such as the activation of epithelial-driven inflammatory pathways involving IL-33, or a too-low eosinophil threshold for enrollment. Physiological eosinophil counts are generally higher during childhood, and eosinophil levels may fluctuate substantially over time due to viral infections, allergen exposure, and developmental immune changes. In the MUPPITS study, the threshold of 150 cells/µL may have resulted in the inclusion of patients whose disease was not predominantly driven by eosinophilic pathways, potentially contributing to the more modest reduction in exacerbations compared with adult studies. In contrast, adolescents generally appear to have benefits similar to adults, as shown in the pooled analyses of the MENSA and MUSCA studies and in pharmacokinetic extrapolation studies supporting regulatory approval. Together, these findings suggest that, while eosinophil depletion is consistently achieved across ages, the relationship between eosinophilic inflammation and clinical outcomes may differ during childhood, highlighting the need for age-specific biomarkers.

Data for benralizumab and depemokimab in children remain comparatively limited, and recommendations on its use cannot be formulated.

Future priorities include adequately powered pediatric trials and real-world comparative effectiveness studies to optimize biologic selection and treatment strategies. Most thresholds, biomarkers, and studies are derived from adults, and predictors of the response to mepolizumab or benralizumab are not yet established; therefore, validated pediatric biomarkers and agreed long-term monitoring are needed. In addition, since airway cellularity (eosinophilic, neutrophilic, mixed, and paucigranulocytic) can change over time [80], longitudinal studies are required to assess the response to biologics in the long term, especially in non-responder subjects.

## Figures and Tables

**Table 1:** Biologics available for children with severe asthma. SCS, systemic oral corticosteroids; FeNO, fraction exhaled nitric oxide; FEV_1_, forced expiratory volume in the first second; TSLP, thymic stromal lymphopoietin; sc, subcutaneous injection; iv, intravenous infusion.

Monoclonal Antibody	Targeted Molecular Mechanism	Effects	Eligibility	Dosage Schedule	Dosage and Administration Method	Age Licensed
Omalizumab	IgE	Reduces IgE-driven allergen response	Severe asthma Serum IgE 30–1500 IU/mL Sensitization to at least one perennial aeroallergen ≥4 courses of SCS in the last 12 months	Every 2–4 weeks depending on IgE level	Dose determined by baseline serum IgE and body weight sc Pre-filled syringe or pre-filled pen	≥6 years
Mepolizumab	IL-5	Inhibits eosinophilic inflammation	Severe eosinophilic asthma (a) Blood eosinophil count ≥300 cells/µL in the last 12 months + ≥4 attacks requiring SCS in the previous 12 months OR (b) Blood eosinophil count ≥400 cells/µL in the last 12 months + at least 3 attacks requiring SCS during the previous 12 months OR daily SCS (prednisolone 5 mg/day) over the previous 6 months	Every 4 weeks. pre-filled syringes or pen	6–11 years: 40 mg ≥12 years: 100 mg sc Pre-filled syringe or pre-filled pen	≥6 years
Benralizumab	IL-5R	Induces apoptosis in eosinophils and basophils	Severe eosinophilic asthma Blood eosinophil count ≥300 cells/µL ≥2 attacks in the last 12 months requiring SCS Reduced FEV_1_ at baseline	Every 4 weeks for the first 3 doses and every 8 weeks thereafter	≥35 kg: 30 mg <35 kg: 10 mg sc Pre-filled syringe or pre-filled pen	≥12 years
Reslizumab	IL-5	Inhibits eosinophilic inflammation	Severe eosinophilic asthma Blood eosinophil count ≥400 cells/µL ≥1 attack in the last 12 months requiring SCS	Every 4 weeks	3 mg/kg iv in healthcare setting	≥18 years
Depemokimab	IL-5	Inhibits eosinophilic inflammation	Severe eosinophilic asthma Blood eosinophil count ≥150 cells/µL ≥1 attack in the last 12 months requiring SCS	Every 6 months	100 mg sc	≥12 years
Dupilumab	IL-4R	Inhibits IL-4 and IL-13 signaling	Severe Th-2 mediated asthma Blood eosinophil ≥150 cells/µL OR FeNO ≥ 25 ppb ≥4 asthma attacks in the last 12 months	Every 2 weeks	6–11 years: dose calculated by body weight ≥12 years: single initial dose of 400 mg, followed by 200 mg every 2 weeks sc Pre-filled syringe or pre-filled pen	≥6 years
Tezepelumab	TSLP	Blocks epithelial inflammatory cascade	Severe uncontrolled asthma No biomarker required ≥3 attacks in the last 12 months	Every 4 weeks	210 mg sc Pre-filled syringe or pre-filled pen	≥12 years

**Table 2:** Table of clinical trials conducted in children with mepolizumab. n, number of participants; FeNO, fractional exhaled nitric oxide; FEV_1_, forced expiratory volume in 1 s.

Author (s), Year	Title	Study Type	Number of Patients (Age)	Aim	Main Results	Limitations
Clinical trials						
Pavord ID, et al., 2012 [41]	Mepolizumab for severe eosinophilic asthma (DREAM): a multicenter, double-blind, placebo-controlled trial	Multicenter, double-blind, placebo-controlled randomized controlled trial.	n = 620 (18–74 yrs) n = 1 (12–17 yrs)	To evaluate the efficacy and safety of mepolizumab in patients with severe eosinophilic asthma, and to identify clinical and biological characteristics associated with treatment response. Specifically, the study aimed to determine the effect of different intravenous doses of mepolizumab on the rate of clinically significant asthma exacerbations, as well as on secondary outcomes including eosinophilic inflammation, lung function, asthma control, and health-related quality of life.	In this multicenter randomized controlled trial, mepolizumab significantly reduced the rate of clinically significant asthma exacerbations compared with placebo across all tested doses (75 mg, 250 mg, and 750 mg), with reductions ranging from 39% to 52%. Treatment also prolonged the time to first exacerbation and reduced exacerbations requiring hospital admission or emergency department visits. Mepolizumab induced a marked and sustained reduction in blood eosinophil counts, with a dose–response relationship, and reduced sputum eosinophils in a subset of patients. However, improvements in lung function (FEV_1_), asthma control, and health-related quality of life were modest and did not consistently reach statistical significance compared with placebo. The safety profile was comparable to placebo, with similar rates of adverse and serious adverse events across groups, and no treatment-related deaths were reported.	The study population was highly selected, including only patients with severe eosinophilic asthma and specific biomarkers of eosinophilic inflammation, which may limit generalizability to the broader asthma population. The inclusion of indirect markers of eosinophilia (e.g., blood eosinophils and FeNO) rather than uniform sputum eosinophil assessment may have introduced heterogeneity in patient characterization. Although the trial was adequately powered for exacerbation outcomes, it was not specifically designed to detect differences in secondary endpoints such as lung function, asthma control, or quality of life. In addition, the duration of follow-up (52 weeks) may be insufficient to fully assess long-term safety and sustained efficacy. Finally, pediatric data were limited (only 1 adolescent included), as the study predominantly included adult patients, restricting the applicability of findings to children and adolescents.
Ortega HG, et al., 2014 [42]	Mepolizumab treatment in patients with severe eosinophilic asthma (MENSA)	Multicenter, randomized, double-blind, placebo-controlled, Phase 3 clinical trial.	n = 551 (50 yrs) n = 25 (12–17 yrs).	To evaluate the efficacy and safety of mepolizumab, administered intravenously or subcutaneously, in reducing exacerbation rates and improving asthma control in patients with severe eosinophilic asthma inadequately controlled with high-dose inhaled glucocorticoids.	Mepolizumab (both intravenous and subcutaneous) significantly reduced the annualized rate of clinically significant exacerbations by approximately 47–53% compared with placebo (*p* < 0.001). Treatment was also associated with modest but significant improvements in lung function (FEV_1_), asthma control (Asthma control questionnaire, ACQ-5), and health-related quality of life (St George’s Respiratory Questionnaire, SGRQ). The incidence of serious adverse events was lower in the mepolizumab groups compared with placebo, with an overall safety profile comparable to placebo.	Limited duration of follow-up (32-week treatment phase) precluding assessment of long-term efficacy and safety; restricted generalizability due to selective inclusion of patients with eosinophilic phenotype and frequent exacerbations; limited data on outcomes after treatment discontinuation; potential sponsor-related bias (industry-funded study with involvement in design and data analysis); and hierarchical statistical testing limiting formal interpretation of some secondary outcomes.
Bel EH, et al., 2014 [43]	Oral glucocorticoid-sparing effect of mepolizumab in eosinophilic asthma (SIRIUS)	Multicenter, randomized, double-blind, placebo-controlled, parallel-group clinical trial (Phase 3).	n = 133 (18–70 yrs) n = 2 (12–17 yrs)	To evaluate the glucocorticoid-sparing efficacy and safety of subcutaneous mepolizumab in patients with severe eosinophilic asthma requiring maintenance oral glucocorticoid therapy, while maintaining asthma control.	Mepolizumab significantly increased the likelihood of achieving a reduction in oral glucocorticoid dose compared with placebo (odds ratio 2.39; *p* = 0.008), with a median dose reduction of 50% versus no reduction in the placebo group (*p* = 0.007). Despite glucocorticoid tapering, mepolizumab reduced the annualized exacerbation rate by 32% (*p* = 0.04) and significantly improved asthma control (ACQ-5) and health-related quality of life (SGRQ). The safety profile was comparable to placebo.	Relatively short study duration, limiting assessment of long-term sustainability of glucocorticoid reduction and clinical outcomes; cautious and protocol-driven glucocorticoid tapering strategy, potentially underestimating the full steroid-sparing effect; assumption that symptom worsening reflects eosinophilic inflammation, which may not apply to all patients; and limited generalizability due to selection of patients with severe eosinophilic asthma requiring maintenance oral glucocorticoids.
Gupta A, et al., 2019 [44]	Subcutaneous mepolizumab in children aged 6 to 11 years with severe eosinophilic asthma	Multinational, non-randomized, open-label, repeat-dose Phase II clinical trial designed to evaluate pharmacokinetics and pharmacodynamics in a pediatric population.	n = 36 (6–11 yrs)	To characterize the pharmacokinetic and pharmacodynamic profile of subcutaneous mepolizumab in children aged 6–11 years with severe eosinophilic asthma, and to compare bodyweight-adjusted drug exposure and eosinophil response with historical adult data, while also exploring preliminary clinical effectiveness and safety.	In 36 children treated with weight-adjusted subcutaneous mepolizumab (40 mg or 100 mg), systemic exposure (AUC) was higher and apparent clearance lower than predicted from adult data, although exposure remained within approximately twofold of target adult levels. Treatment induced marked and sustained reductions in blood eosinophil counts (−88.5% and −83.4% at week 12 in the 40 mg and 100 mg groups, respectively). Improvements in asthma control scores (ACQ-7 and C-ACT) were observed, although lung function changes were not consistent. The safety profile was favorable, with no new safety signals and tolerability comparable to that reported in adults and adolescents.	The small sample size, exploratory nature of clinical endpoints, and limited follow-up duration necessitate cautious interpretation of efficacy magnitude and long-term outcomes.
Jackson DJ, et al., 2022 [45]	Mepolizumab for urban children with exacerbation-prone eosinophilic asthma in the USA (MUPPITS-2): a randomized, double-blind, placebo-controlled, parallel-group trial	Randomized, double-blind, placebo-controlled, parallel-group clinical trial.	n = 290 (6–17 yrs)	To evaluate the efficacy and safety of subcutaneous mepolizumab, compared with placebo, in reducing asthma exacerbations and improving clinical outcomes in children with severe eosinophilic asthma.	Mepolizumab significantly reduced the annualized rate of clinically significant asthma exacerbations compared with placebo and achieved a substantial and sustained reduction in blood eosinophil counts. Improvements were also observed in asthma control and quality-of-life measures, while lung function changes were modest. The safety profile was comparable to placebo, with no increase in serious adverse events.	Limited pediatric sample size and restricted age range, reducing generalizability; relatively short treatment and follow-up duration for long-term outcomes; exclusion of patients with significant comorbidities, potentially limiting external validity; modest effects on lung function endpoints; and limited statistical power for some secondary and subgroup analyses.
Chupp GL et al., 2017 [46]	Efficacy of mepolizumab add-on therapy on health-related quality of life and markers of asthma control in severe eosinophilic asthma (MUSCA): a randomized, double-blind, placebo-controlled, parallel-group, multicenter, phase 3b trial	Multicenter, randomized, double-blind, placebo-controlled, parallel-group, Phase 3 clinical trial.	n = 542 (18–82 yrs) n = 9 (12–17 yrs)	To assess the effect of mepolizumab on disease-specific health-related quality of life (HRQOL) using the St George’s Respiratory Questionnaire (SGRQ) as the primary endpoint in patients with severe eosinophilic asthma. Secondary endpoints included the mean change from baseline in pre-bronchodilator FEV_1_, proportion of SGRQ responders (≥4-point reduction), and mean change in asthma control (ACQ-5 score).	Mepolizumab significantly improved SGRQ total score versus placebo at week 24 (change: −15.6 vs. −7.9; difference −7.7, *p* < 0.0001), exceeding the MCID. Pre-bronchodilator FEV_1_ increased more with mepolizumab (+176 mL vs. +56 mL; *p* = 0.001), as did ACQ-5 responders (59% vs. 42%; OR 2.0, *p* = 0.0014). Clinically significant exacerbations were reduced by 56% (rate ratio 0.56, *p* < 0.0001); safety profile was similar to placebo.	Placebo group SGRQ improvements exceeded MCID at week 24, possibly due to enhanced adherence under trial monitoring. Short 24-week treatment duration limits assessment of long-term effects. Exclusion of current smokers and high FEV_1_ reversibility may restrict generalizability.
Other studies on children						
**Author (s), Year**	**Title**	**Study Type**		**Aim**	**Main Results**	**Limitations**
Yancey SW, et al., 2019 [47]	Efficacy of add-on mepolizumab in adolescents with severe eosinophilic asthma	Post hoc pooled subgroup analysis of multicenter, randomized, double-blind, placebo-controlled Phase II/III clinical trials.		To evaluate the efficacy and safety of add-on mepolizumab in adolescents (12–17 years) with severe eosinophilic asthma, through a subgroup and pooled post hoc analysis of data derived from Phase II/III randomized controlled trials.	Add-on mepolizumab in adolescents with severe eosinophilic asthma was associated with a clinically meaningful reduction in the annual rate of exacerbations (~40% in pooled analyses) and a marked decrease in blood eosinophil counts, with effects consistent with those observed in the adult population. Pharmacokinetic profiles were comparable between adolescents and adults. The safety profile was favorable and aligned with that reported in the overall study populations, with no treatment-related serious adverse events and no study withdrawals due to adverse events.	Small sample size of the adolescent subgroup, limiting statistical power and precision of estimates; post hoc subgroup and pooled analyses not specifically designed to assess efficacy in adolescents; absence of formal statistical comparisons in this age group; heterogeneity across included Phase II/III trials (different designs, durations, and dosing regimens); and potential selection bias related to low recruitment of adolescents with severe eosinophilic asthma.
Gupta A, et al. 2026 [48]	Development of Pediatric Dosing for Mepolizumab in Severe Asthma Based on Extrapolation of Data from Adult Patients and a Phase II Open-Label Pediatric Trial	Multimethod pharmacometric study based on partial extrapolation of adult Phase III randomized controlled trial data combined with a Phase II open-label pediatric trial, incorporating population pharmacokinetic/pharmacodynamic (PK/PD) modeling and meta-analysis.		To develop and validate pediatric dosing recommendations for mepolizumab in severe eosinophilic asthma by applying a partial extrapolation strategy integrating adult Phase III efficacy and safety data with pediatric pharmacokinetic/pharmacodynamic evidence from an open-label Phase II trial.	Partial extrapolation demonstrated that mepolizumab pharmacokinetics and pharmacodynamics are consistent across age groups, with body weight as the main determinant of drug exposure. Pediatric patients showed comparable blood eosinophil reduction (≈83–92%) and similar clinical outcomes to adults and adolescents, with no new safety signals identified. Model-based analyses supported equivalent efficacy between adolescents and adults, and consistent responses in children. These findings enabled selection of weight-adjusted dosing (40 mg SC in children <12 years; 100 mg SC in adolescents), achieving exposure comparable to adults and supporting regulatory approval.	Limited pediatric sample size, particularly in the open-label Phase II trial (n= 36), and very low representation of adolescents in pivotal randomized controlled trials, restricting direct efficacy comparisons. Absence of a placebo-controlled arm and short treatment duration (12 weeks) in the pediatric study limit robust efficacy assessment in children. Reliance on model-based extrapolation and assumptions of similar disease pathophysiology and exposure–response relationships across age groups may introduce uncertainty. Safety analyses were primarily descriptive and partly derived from heterogeneous populations and indications.
Gupta A, et al. 2019 [49]	Long-term safety and pharmacodynamics of mepolizumab in children with severe asthma with an eosinophilic phenotype	Open-label, uncontrolled, single-arm, repeat-dose extension study (Phase II extension trial).		To evaluate the long-term safety, pharmacodynamics, and exploratory efficacy of subcutaneous mepolizumab in children aged 6–11 years with severe eosinophilic asthma.	Mepolizumab demonstrated a favorable long-term safety profile, with no treatment-related serious adverse events and no new safety signals identified over 52 weeks. Treatment resulted in sustained reductions in blood eosinophil counts and was associated with improvements in asthma control scores and a marked decrease in annualized exacerbation rates compared with baseline.	Open-label, uncontrolled design without a comparator group; small sample size limiting statistical power and generalizability; absence of randomization and blinding; reliance on exploratory efficacy analyses with indirect comparison to adult/adolescent studies; limited post-treatment follow-up in patients entering the long-term access program.
Altman MC et al., 2025 [50]	Inflammatory Pathways in Residual Asthma Exacerbations Among Mepolizumab-Treated Urban Children: A Secondary Analysis of a Randomized Clinical Trial	Secondary analysis of a Phase 2 randomized clinical trial (double-blind, placebo-controlled, parallel-group).		To identify multiple distinct molecular mechanisms implicated in asthma exacerbations by characterizing respiratory illnesses among urban children with eosinophilic asthma enrolled in a clinical trial comparing treatment with mepolizumab vs. placebo.	In mepolizumab-treated children, exacerbations showed blunted type 2 (T2) inflammation (eosinophil module log_2_FC −0.60, FDR < 0.05) but upregulated epithelial (e.g., Smad3 differentiation log_2_FC 0.33; cilia/IL-33 log_2_FC 0.82) and macrophage (e.g., chemoattraction log_2_FC 0.38) modules versus placebo-treated exacerbations (all FDR < 0.05); mucus hypersecretion and stress responses were elevated in all exacerbations; three semiorthogonal inflammatory axes explained 78.5% heterogeneity.	Secondary analysis design precludes causal inferences regarding molecular pathways; observational nasal sampling during illnesses may introduce selection bias; reliance on predefined transcriptomic modules limits detection of novel genes/pathways; lack of mechanistic validation precludes functional causality; generalizability constrained to urban, eosinophilic asthma phenotypes in mepolizumab-treated children
Liu NM et al., 2025 [51]	Severe Paediatric Asthma Collaborative in Europe: real-world data on children on biologics	Multicenter, observational, cross-sectional real-world study based on a European registry (SPACE cohort).		To characterize clinical features, asthma control, lung function, exacerbations, quality of life, and patterns of biologic use (including switching) in children and adolescents with severe asthma receiving biologic therapies, and to identify unmet clinical needs in this population.	In a cohort of 250 pediatric patients (median age 13.2 years), most children receiving biologics (omalizumab, mepolizumab, or dupilumab) achieved good asthma symptom control (64.8%) and satisfactory quality of life. However, a substantial proportion remained partly controlled (26.8%) or uncontrolled (8.4%), with persistent disease burden. Over the preceding 12 months, 51.6% experienced ≥1 exacerbation and 28.8% ≥2 exacerbations; 9.6% required hospitalization. Airflow obstruction persisted in a relevant subset (≈33%). Switching between biologics occurred in 16% of patients, predominantly due to lack of efficacy. Overall, despite biologic therapy, significant residual morbidity and unmet therapeutic needs were observed.	The cross-sectional design precludes evaluation of longitudinal changes and causal inference regarding treatment effects. Lack of pre-biologic baseline data limits assessment of treatment response. Biomarkers such as eosinophils and atopy may be influenced by ongoing biologic therapy, reducing their interpretability. Heterogeneity in treatment selection across centers may introduce confounding. Additionally, real-world data collection may be subject to variability in clinical practice and measurement, and longitudinal follow-up data are not yet available.

**Table 3:** Clinical effects of mepolizumab in children and adolescents with severe eosinophilic asthma.

Clinical Effect	Key Findings	References
Reduction in exacerbations	Reduction ranging from 27–69% across RCTs and real-world studies; 53% reduction in MENSA, 32% in SIRIUS, 39–52% in DREAM, and 69% in the study by Gupta et al.	[41,42,43,45,47,49,58,59]
Reduction in blood eosinophils	86% reduction in MENSA trial; from 70 to 90% in DREAM and MUPPITS-2 trials, respectively.	[41,42,44,45]
Improvement in asthma symptoms and control	Improved symptom scores and asthma control reported in MENSA, SIRIUS, MUSCA trials, and pediatric studies.	[42,43,44,46]
Improvement in quality of life	Significant improvements in health-related quality of life in MENSA, SIRIUS, MUSCA trials, and pediatric cohorts.	[42,43,44,46,49]
Improvement in lung function	Modest increase in FEV_1_ (~100 mL) in MENSA trial.	[42]
Reduction in oral corticosteroid use	Up to 50% reduction in maintenance OCS dose in SIRIUS; 24% reduction in real-world pediatric data.	[43,58]
Reduction in hospitalizations	Hospital admissions decreased by 67% after initiation of mepolizumab in a retrospective pediatric cohort.	[59]

**Table 4:** Table of studies including children on benralizumab.

Author (s), Year	Title	Study Type	Number of Patients (Age)	Aim	Main Results	Limitations
Wedner et al., 2024 [70]	Benralizumab in children with severe eosinophilic asthma: Pharmacokinetics and long-term safety (TATE study)	Phase 3, 48-week, open-label, parallel-group, multicenter.	n = 28 (6–11 yrs)	To evaluate pharmacokinetics, pharmacodynamics, safety, and tolerability of benralizumab in children (6–11 years) with severe eosinophilic asthma.	Benralizumab showed pharmacokinetics comparable to adults and adolescents; effectively reduced blood eosinophils to near-zero; generally well-tolerated with no new safety signals; improvements observed in asthma control and exacerbation rates.	Open-label design (no control group); relatively small sample size; limited power for efficacy outcomes; short duration for long-term conclusions despite extension; lack of placebo comparison.
Bleecker et al., 2016 [71]	Efficacy and safety of benralizumab for patients with severe asthma uncontrolled with high-dosage inhaled corticosteroids and long-acting β2-agonists (SIROCCO)	Phase 3, 48-week, randomized, double-blind, parallel-group, placebo-controlled, multicenter.	n = 1152 (18–75 yrs) n = 53 (12–17 yrs)	To evaluate efficacy and safety of benralizumab as add-on therapy in severe uncontrolled eosinophillic asthma.	Significant reduction in annual asthma exacerbation rates; improved lung function (FEV_1_); good safety profile comparable to placebo.	Limited duration (48 weeks); mainly eosinophilic phenotype; generalizability to broader asthma populations uncertain.
FitzGerald et al., 2016 [72]	Anti–interleukin-5 receptor α monoclonal antibody as add-on treatment for severe uncontrolled eosinophilic asthma (CALIMA)	Phase 3, 56-week, randomized, double-blind, parallel-group, placebo-controlled, multicenter.	n = 1251 (18–75 yrs) n = 55 (12–17 yrs)	To assess efficacy and safety of benralizumab in reducing exacerbations in severe asthma.	Reduced exacerbation rates (especially in high eosinophil counts); modest improvement in lung function; well-tolerated.	Smaller effect size vs. SIROCCO; variability in response; short follow-up.
Busse et al., 2019 [73]	Long-term safety and efficacy of benralizumab: 1-year results from BORA phase 3 extension trial	Phase 3, randomized, double-blind, parallel-group extension study of SIROCCO AND CALIMA (56 weeks for adults; 108 weeks for adolescents).	n = 1836 (12–75 yrs) n = 90 (12–17 yrs)	To evaluate long-term safety and sustained efficacy of benralizumab.	Sustained reduction in exacerbations; maintained/improved lung function; no new safety signals.	Open-label extension; potential bias; no placebo control.
Zeitlin et al., 2018 [74]	Benralizumab does not impair antibody response to seasonal influenza vaccination (ALIZE)	Phase 3b, 12-week, randomized, double-blind, parallel-group, placebo-controlled, multicenter.	n = 103 (12–21 yrs)	To assess whether benralizumab affects immune response to influenza vaccine.	No impairment in antibody response; vaccine remained effective; supports immunological safety.	Limited sample size; specific age group (adolescents/young adults); short-term immunogenicity only.

## Data Availability

No new data were created or analyzed in this study.

## Supplementary Materials

The following supporting information can be downloaded at: https://www.mdpi.com/article/10.3390/cells15141246/s1, Figure S1: literature search and study selection process.
