# Supplementary material for: Monoclonal Antibodies Directed Against IL-5 in the Treatment of Pediatric Asthma
Source: Cells. 2026 Jul 10;15(14):1246. doi: 10.3390/cells15141246 (PMC13407027; doi:10.3390/cells15141246)
Supplement: Supplementary file 1 [file cells-15-01246-s001.zip › cells-4323127-supplementary.pdf]

Supplementary methods

Records identified on Scopus with the following search strategies (limits: English, Article, Human, Child, Adolescent):

"mepolizumab AND children AND severe asthma": 72

"benralizumab AND children AND severe asthma": 36

"depemokimab AND children AND severe asthma": 1

Records identified on Pubmed with the following search strategies (limits: English, age 0-18 years):

"mepolizumab AND severe asthma": 115

"benralizumab AND severe asthma": 65

"depemokimab AND severe asthma": 1

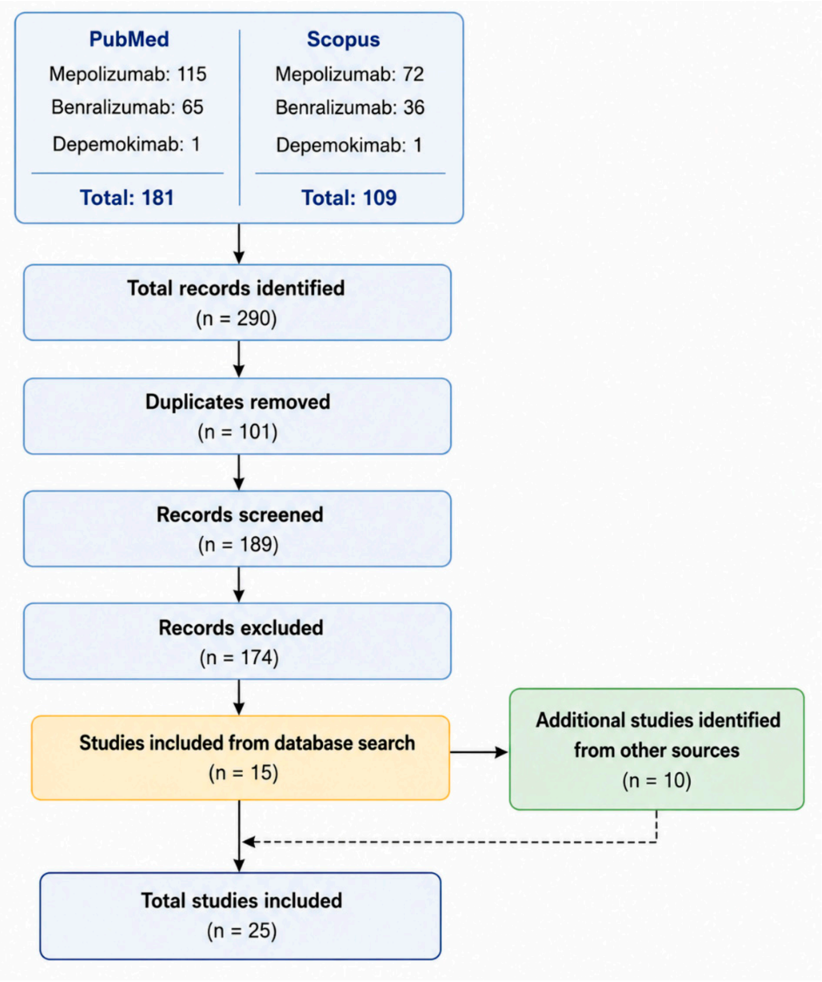

Figure S1. Literature search and study selection process.
